# Bat pathogens hit the road: But which one?

**DOI:** 10.1371/journal.ppat.1007134

**Published:** 2018-08-09

**Authors:** Léa Joffrin, Muriel Dietrich, Patrick Mavingui, Camille Lebarbenchon

**Affiliations:** Université de La Réunion, Processus Infectieux en Milieu Insulaire Tropical, INSERM 1187, CNRS 9192, IRD 249, Saint Denis, Réunion Island, France; University of Kentucky, UNITED STATES

Current evidence suggests that environmental changes and interactions between wildlife, livestock, and humans contribute to the spillover of infectious agents from bats to other hosts [1]. An increasing number of studies investigating the diversity and infection dynamics of bat pathogens has recently been published; however, how these infectious agents are transmitted both within bat populations and to other hosts, including humans, often remains unknown. Here, we summarize current knowledge on direct and indirect transmission routes of bat infectious agents of public health concern (Fig 1). Although bats are recognized as major reservoir hosts of emerging infectious diseases, we highlight that a significant knowledge gap on transmission mechanisms remains and needs further exploration.

**Fig 1:**
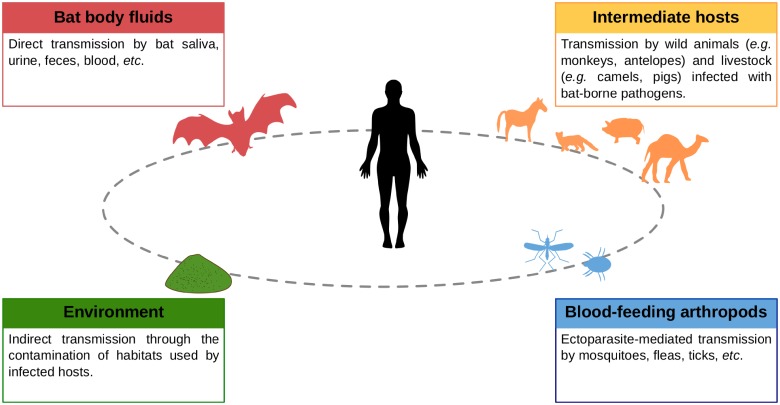
Possible transmission routes of bat-borne pathogens to humans.

## Bat bites: The exception rather than the rule?

Although bat bites may be the main transmission route coming to mind, pathogen transmission involving bat bites has been documented mostly for rabies virus (Rhabdoviridae). The common vampire bat (*Desmodus rotundus*) can, for instance, naturally transmit rabies to other species when biting to feed on blood, particularly to livestock and sometimes to humans [2]. *Mycoplasma* has also been detected in common vampire bat blood and saliva and might be transmitted between bats, for instance, during aggressive behaviors [3]. Obligate blood-feeding bats are, however, restricted to Central and South America and represent only a very small proportion of the bat species diversity (<0.005%; 3/1,200). Most bat species do not naturally bite humans unless intentional contacts occur (e.g., veterinarian and field biologists involved in bat capture and handling, people trying to remove bats from houses).

Contact with bat body fluids (saliva, urine, and feces) is increasingly recognized as an important mechanism of pathogen spillover to humans. Human encroachment into bat habitats as well as increasing urbanization, which facilitates bat roosting in artificial structures, are likely to increase contact with bat body fluids. For example, Nipah virus (Paramyxoviridae) human infection cases reported in Bangladesh were associated with the consumption of raw sap from date palm trees contaminated with fruit bat saliva and urine [4]. In the case of Marburg virus (Filoviridae), experimental studies indicate that bat-to-bat transmission may occur via saliva and aerosols, suggesting that the virus may be transmitted to other hosts by a similar mechanism [5,6]. This hypothesis is supported by investigations revealing that most humans infected with Marburg virus had entered bat (*Rousettus aegyptiacus*) caves before becoming sick and reported regular contacts with bats or their secretions [7].

Hunting, preparation, and consumption of bats as bushmeat have also been pointed out as a potential source of infection, especially for Ebola virus. For instance, the putative first human case of the 2007 Ebola outbreak in the Democratic Republic of Congo would have bought freshly killed bats for consumption [8]. The fruit bat *Eidolon helvum*, which is the most frequently hunted and traded bat species in many African countries (e.g., more than 120,000 *E*. *helvum* are sold yearly in markets in Ghana [9]), has been shown to be infected with Henipa-related viruses. This highlights the substantial exposure of local hunters and consumers to viruses of potential zoonotic importance [10].

## Can other animals transmit bat-borne pathogens?

Infection of wild animals such as apes, monkeys, and antelopes with bat-borne infectious agents may also play a role in the transmission chain to humans, such as for Ebola virus [11]. In the case of the severe acute respiratory syndrome (SARS) coronavirus, civets (*Paguma larvata*) got infected with a virus circulating in horseshoe bats (*Rhinolophus* sp.) and would have then acted as an intermediate amplifying host [12]. Natural bat predation by other animals (e.g., monkeys, domestic cats) and its consequences on infectious agents transmission are poorly documented [13,14] but could also favor spillover opportunities to other hosts.

In addition to wild animals, the role of livestock as intermediate and amplifying hosts between wild animals and humans has been clearly demonstrated in several outbreaks, such as for Filovirus and Henipavirus [15,16]. Indeed, in Malaysia, the growth of commercial pig farms with fruit trees on the farm has created an environment where bats could drop partially eaten fruits contaminated with Nipah virus into pig stalls [4,17].

In contrast to rapid and short-time spillover events, long-time and silent circulation of viruses in livestock before transmission to humans may also occur, as is strongly suspected for the ongoing outbreak of Middle East respiratory syndrome (MERS). Although bats are likely to be a source of MERS-like coronaviruses, dromedary camels (*Camelus dromedaries*) act as the natural reservoir host in which the MERS coronavirus could have circulated for more than 30 years before its first detection in humans [18]. Other animals such as llamas (*Lama glama*) and wild boars (*Sus scrofa*) have shown susceptibility to MERS coronavirus infection [19], suggesting a large host species range. The endemic human coronavirus 229E may also constitute a descendant of camelid-associated viruses [20] and further supports that livestock plays a key role in the long-time establishment of bat-borne viruses in humans.

## Are blood-feeding arthropods involved in bat-borne pathogen transmission?

A large diversity of arthropods, such as mosquitoes, mites, flies, fleas, and ticks [21], can be found in habitats occupied by bats, particularly in cave systems. Some bat ectoparasites (e.g., fleas and ticks) might incidentally bite humans [22], but ectoparasite-mediated transmission of bat-borne infectious agents to humans is difficult to demonstrate and has rather been speculated, such as for the transmission of Ebola virus [23]. Nevertheless, the presence of the bacterium *Bartonella mayotimonensis*, the etiologic agent of endocarditis in humans, both in bat blood and fleas, suggests that transmission to humans by flea bites or their fecal droppings may occur [24]. With the advance of metagenomic technologies, a large diversity of potentially zoonotic bacteria (e.g., *Rickettsia*, *Bartonella* [25]) have been described in bat ectoparasites, but such investigations remain scarce for other infectious agents, such as haemosporidian parasites and viruses [26]. Dengue virus (DENV) was recently detected simultaneously in bat flies (Streblidae) and in their host (*Desmodus rotundus*), although DENV transmission from bat flies to humans has never been reported [27]. To date, the role of blood-feeding arthropods in pathogen spillover to humans therefore remains highly speculative.

## Does environmental transmission occur?

Environmental transmission or indirect transmission through the contamination of habitats used by infected hosts has been described as a major mechanism in the epidemiology of wildlife diseases. For bat infectious agents, a limited number of experimental and field studies have been performed to assess their persistence in the environment. Henipaviruses can persist in liquids and on solid surfaces for several days under laboratory conditions and filoviruses for several weeks [28,29]. Bat-borne *Leptospira* could be a source of contamination to other hosts, as this bacterial genus is known to persist in moist environments [30]. Transmission may also occur by bat guano (i.e., accumulation of bat excrement in the environment). Indeed, guano from cave-dwelling bats is commonly used in agriculture as fertilizer worldwide [31]. Reports of human infection with bat guano are usually restricted to histoplasmosis, also known as “cave disease,” a lung infection caused by a fungus (*Histoplasma capsulatum*). The detection of coronavirus RNA in bat guano has been demonstrated [32], although there was no evidence of long-time maintenance of infectious viral particles by virus isolation. Longitudinal sampling of environmental material (water, guano, and soil) exposed to bat secretions for the detection, quantification, and isolation of infectious agents is needed to better assess the risk associated with this transmission route.

## What can we learn from bat-to-bat transmission?

Transmission of infectious agents is highly dynamic in bats and is associated with significant changes in bat population structure (e.g., birth pulse in maternity colonies) [1]. Periods of high prevalence of infected bats with Hendra and Marburg viruses have been shown to coincide with the timing of infectious agent spillover to other hosts [1,33]. Although several studies have focused on these aspects, a precise assessment of the diversity of transmission routes involved in disease epidemiology in bats is still lacking, especially when considering the extreme diversity of bat species and associated ecological and biological features. Such information is not only relevant from a fundamental perspective but can provide major information for the development of biosafety measures, therefore limiting emergence risk.

Although communication and education on the risk associated with bat-borne pathogens has increased over the past decade, the benefits of bats in ecosystem functions (e.g., pollination, soil fertilization, and crops pest control) tend to be disregarded [34]. Gaining knowledge on disease epidemiology and bat ecology is critical to fully assess the challenges associated with human health and bat conservation. In this context, implementation of One Health approaches seems essential and beneficial for a sustainable development, particularly for populations living in hotspots of bat-borne disease emergence [35].
